# Enhancing elderly care: Efficient and reliable real-time fall detection algorithm

**DOI:** 10.1177/20552076241233690

**Published:** 2024-02-20

**Authors:** Yue Wang, Tiantai Deng

**Affiliations:** 1 Department of Electronic and Electrical Engineering, 7315 The University of Sheffield, Sheffield, UK

**Keywords:** Biomechanics, human action recognition, image processing, machine learning, pose estimation, smart healthcare

## Abstract

**Background and Objective:**

Falls pose a significant risk to public health, especially for the elderly population, and could potentially result in severe injuries or even death. A reliable fall detection system is urgently needed to recognise and promptly alert to falls effectively. A vision-based fall detection system has the advantage of being non-invasive and affordable compared with another popular approach using wearable sensors. Nevertheless, the present challenge lies in the algorithm's limited on-device operating speed due to extremely high computational demands, and the high computational demands are usually essential to improve the performance for the complex scene. Therefore, it is crucial to address the above challenge in computational power and complex scenes.

**Methods:**

This article presents the implementation of a real-time fall detection algorithm with low computational costs using a single webcam. The suggested method optimises precision and efficiency by synthesising the strengths of background subtraction and the human pose estimation model BlazePose. The biomechanical features, derived from body key points identified by BlazePose, are utilised in a random forest model for classifying fall events.

**Results:**

The proposed algorithm achieves 89.99% accuracy and 29.7 FPS with a laptop CPU on the UR Fall Detection dataset and the Le2i Fall Detection dataset. The algorithm shows great generalisation and robustness in different scenarios.

**Conclusion:**

Due to the low computational power of the system, the findings also suggest the potential for implementing the system in small-scale medical monitoring equipment, which maximises its practical value in digital health.

## Introduction

Falls have become the second leading cause of unintentional injury death worldwide.^1^ And the related concern is growing worse among the elderly population.^2^ Leaving the fallen elderly unattended could be life-threatening; therefore, the autonomous fall detection system is highly significant since it has the ability to provide a timely response to a fall event.

Currently, there are three popular methods being applied to detect falls.^3^ Falls can be determined by measuring the necessary data using wearable sensors, such as gyroscopes and accelerometers. However, this approach cannot work effectively due to the limited battery life of devices and the discomfort when wearing them.^4^ Alternatively, some ambient sensors like radars, RF sensors, and ultrasonic sensors are applied for fall recognition.^3^ Although the method based on ambient sensors provides a decent and non-intrusive solution, the complexity of system installation and maintenance brings troublesome issues. As another non-intrusive fall detection method, the vision-based approach is relatively preferred, offering the advantages of dominant precision and simple system construction.

Most recent studies about the vision-based method have predominantly focused on deep learning techniques for optimal accuracy. The researchers managed to employ deep learning techniques in sub-tasks of fall detection, such as human detection, pose estimation, and fall recognition. The emergence of pose estimation neural networks has significantly boosted the precision of body spatial representation, which can be considered a milestone for action recognition. But these advances compromise computational speed, which leads to the challenges of on-device real-time detection.

**Figure 1.**
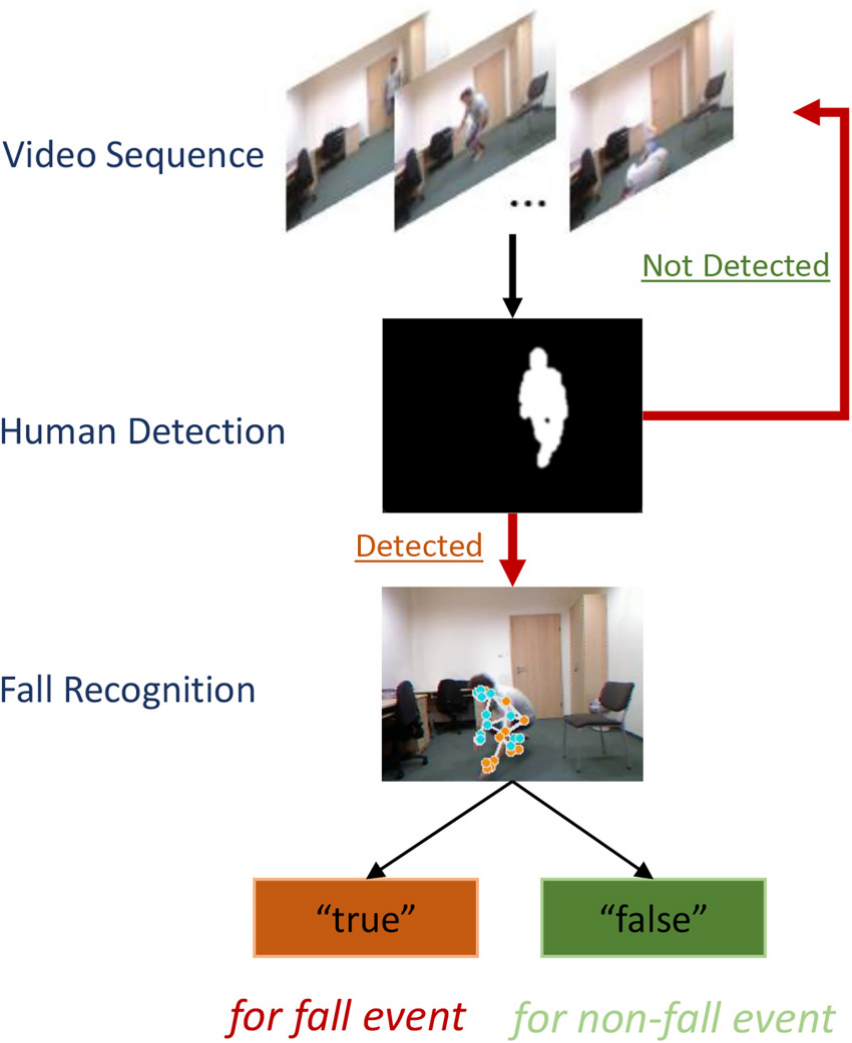
Schematic diagram of the proposed fall detection algorithm.

This paper proposes an improved algorithm based on the RGB input shown in Figure 1 to combine the benefits of digital image processing based on background subtraction and the human pose estimation model. A series of fundamental digital image processing techniques serves as a human detector. Furthermore, the human pose estimation model BlazePose checks for the existence of a moving person and performs fall recognition if a moving person exists in the scene. The suggested algorithm optimises the average computational costs and shows a high level of accuracy with cascaded random forest classifiers. This improvement makes the fall detection system more realistic for real-time performance on embedded systems in compact monitoring devices with low computing power. The main contributions of this paper are as follows:

- We introduce a more efficient and faster fall detection algorithm with high reliability. We obtained a remarkable accuracy of 89.99%, along with a real-time performance at 29.7 FPS on a laptop CPU.
- The proposed algorithm requires a simple and low-cost system construction since it only relies on a low-cost web camera, for example, a basic 480p RGB camera, and an affordable device with low computational power for processing, such as a budget laptop with a Ryzen 7 processor.
- We present an explainable fall detection algorithm, which is contributed by the physical significance of the selected features for fall recognition. Furthermore, we obtain an accuracy of approximately 90% on both the UR Fall Detection dataset^5^ and the Le2i Fall Detection dataset.^6^ Since both datasets cover various scenarios, this result also highlights the robustness and effectiveness.

The rest of this paper is structured as follows: next section investigates the related research, section ‘Methodology’ goes through the methodology, section ‘Results and evaluation’ demonstrates the experimental results and compares them with the existing literatures, and section ‘Conclusions’ summarises the findings of the research.

## Related work

Several works related to real-time fall detection algorithms have been proposed to enhance the practical value and reduce the system cost. This section commences with the overview of the state-of-the-art approaches as appraised in the recent survey^7^ and then reviews the work undertaken to pursue the development of real-time fall detection systems.

Keskes and Noumeir^8^ used spatial temporal graph convolutional networks (ST-GCNs) based on skeleton-based features for fall detection. ST-GCN is designed to analyse spatial–temporal data and capture complex patterns in the sequence, which shows improved performance in action recognition.^8^ The skeletal features with 25 key points were extracted by using Microsoft's Kinect v2 sensor. And transfer training was applied to resolve the insufficiency of the data. The suggested approach achieved an accuracy of 100% and 92.91% on the TST v2 dataset and FallFree dataset.

Sun and Wang^9^ proposed a vision-based fall detection algorithm which obtains an accuracy of 94.6%. The features were extracted using OpenPose, which is a powerful pose estimation algorithm with 25 body key points provided based on part affinity fields.^10^ Moreover, the object detection model SSD-MobileNet was applied to remove the interference of non-human objects and raise the confidence of correct body key points. Support vector data description (SVDD) algorithm for action classification was selected to overcome the data imbalance.

Similar to the two aforementioned works, many state-of-the-art studies involve some computationally expensive techniques, such as neural networks for pose estimation and action recognition. They are extremely difficult to operate in real-time without the high-spec computing platforms, and this limitation is detrimental in the fall detection system where the intermediate response is essential. Moreover, the complexity of these systems compromises their simplicity and cost-effectiveness in practical applications.

To solve these challenges, some works have been dedicated to developing real-time fall detection systems. They attempted to optimise the required computation resources in many ways, including lightweight algorithm,^11^ different modalities^12^ and novel computing architecture.^13^

Alam et al.^11^ proposed a real-time fall detection algorithm with low computational power. A lightweight pose estimation model MoveNet was implemented to calculate the position difference between the upper and lower body. A simple threshold-based classification method was used to complete the final predictions. The speed of this designed algorithm on the CPU exceeded 30 FPS, but the accuracy was not high.

Qian et al.^12^ designed a low-cost, real-time fall detection system based on the wearable sensor. The used sensor is a 6-axis motion tracking MEMS sensor with a gyroscope and accelerometer. This study suggested a multi-level thresholding approach on the basis of features derived from axial acceleration, angular velocity and inclination angle. The accuracy rate of 94.88% was obtained in the designed experiment. Due to the algorithm's simplicity and efficient communication on the Internet of Things (IoT) platform, the system efficiency can be very high.

Lin et al.^13^ applied the concept of AI-based edge computing to the vision-based fall detection system and suggested a lightweight algorithm on the Sipeed MAix Go AI development board. YOLO-LW was used to find out the bounding box of the human, and SVM was the final classifier based on features from the contour. It is reported that the edge computing platform reached an accuracy of 91.1% and an operating speed of 11.5 FPS.

The insufficiency of robustness might be one of the main problems among these three studies related to real-time fall detection. The studies conducted by Alam et al.^11^ and Qian et al.^12^ applied the simple threshold-based classifier for fall recognition. The threshold-based classifier strongly depends on the parameter settings, which indicates the algorithm might have lower performance in the different scenes without testing. Besides, the features used lack the necessary diversity in the work of Alam et al.^11^ and Qian et al.,^12^ which means the accuracy of their system might be threatened in complex environments. In terms of system configuration, a large-size embedded sensor was required in the work from Qian et al.,^12^ which might lead to discomfort and inconvenience. The core platform in Lin et al.’s^13^ design was the AI chip, and they pointed out that the debugging is challenging and time-consuming on the AI chip.^13^

Based on the works of literature reviewed above, the current challenges are summarised as follows:

1. Special equipment is required, like Microsoft's Kinect v2 sensor in Keskes and Noumeir^8^ and MEMS sensor in Qian et al.,^12^ which introduce higher design and maintenance expenses.
2. The computing power requirement is too high because of heavyweight neural networks, like ST-GCN in Keskes and Noumeir^8^ and OpenPose in Sun and Wang,^9^ which makes it difficult to achieve real-time detection in low-cost systems.
3. A limited number of biomechanical features reduces the robustness of the proposed design, like Alam et al.^11^ and Lin et al.^13^

In order to tackle the mentioned challenges, this research proposes a low-cost and real-time fall detection system with great reliability and robustness. The whole system is implemented on a normal laptop CPU with a basic webcam, and the system achieves an excellent balance between operational speed and accuracy.

## Methodology

In this section, we are going to introduce our low-cost algorithm for fall detection. All the testing data are from public available dataset (UR Fall Detection^5^ and a portion of the Le2i Fall Detection^6^) thus there is no need for patient's consent in this research.

### Algorithm overview

**Figure 2.**
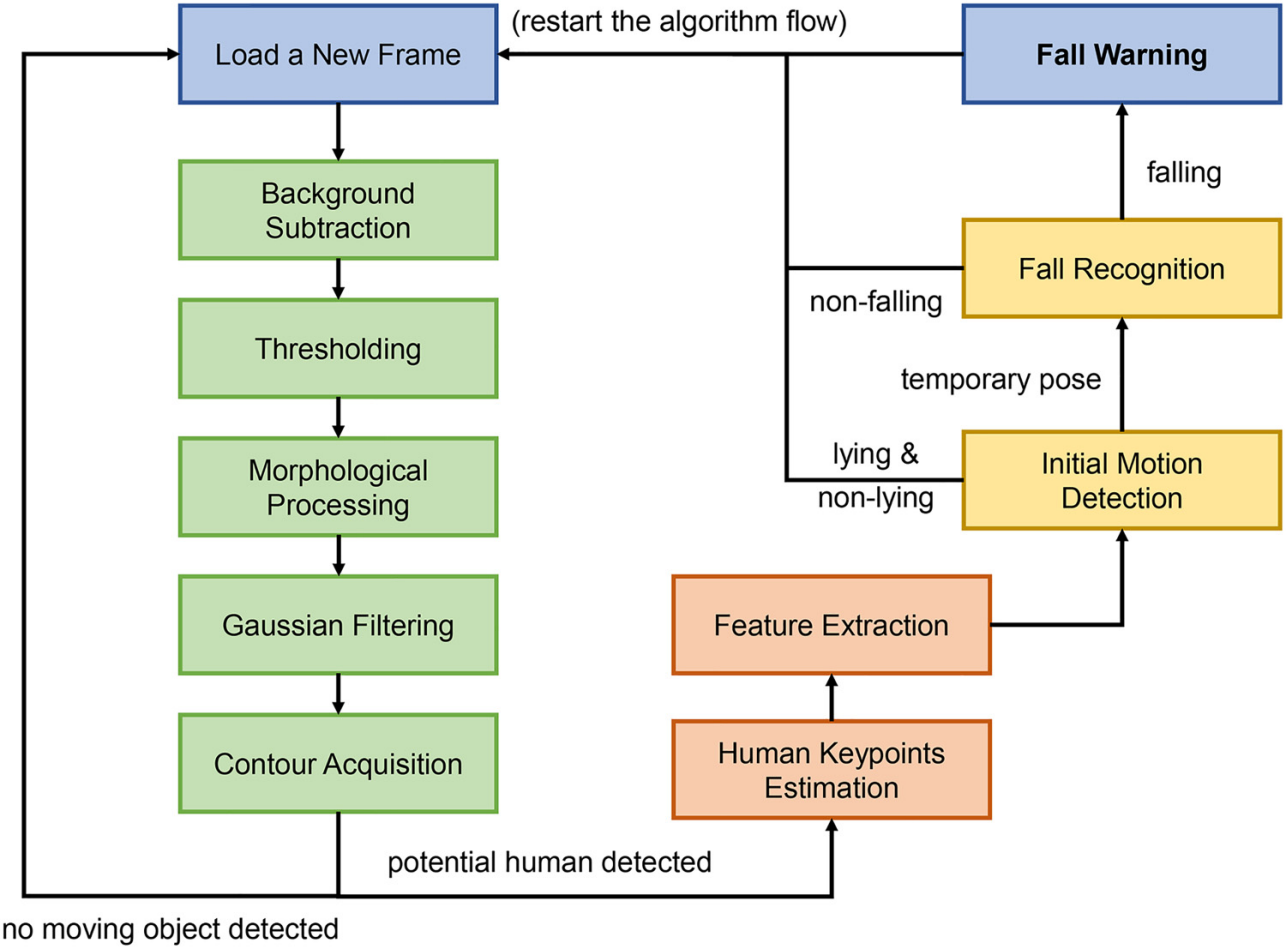
Comprehensive flowchart of the completed vision-based fall detection algorithm. Blue blocks indicate the input and output module, green blocks depict the flow of the human detector, red blocks explain the origin of the features, and yellow blocks represent the classifier component.

We propose a frame-by-frame and efficient processing algorithm. All the critical components and interconnections of the algorithm are covered in Figure 2. Moreover, we provide two conditional options to end the processing of the current frame in advance when the algorithm cannot identify the current frame as a potential fall event. For example, no moving person exists, or a person keeps standing in the scene.

### Hybrid human detector

**Figure 3.**
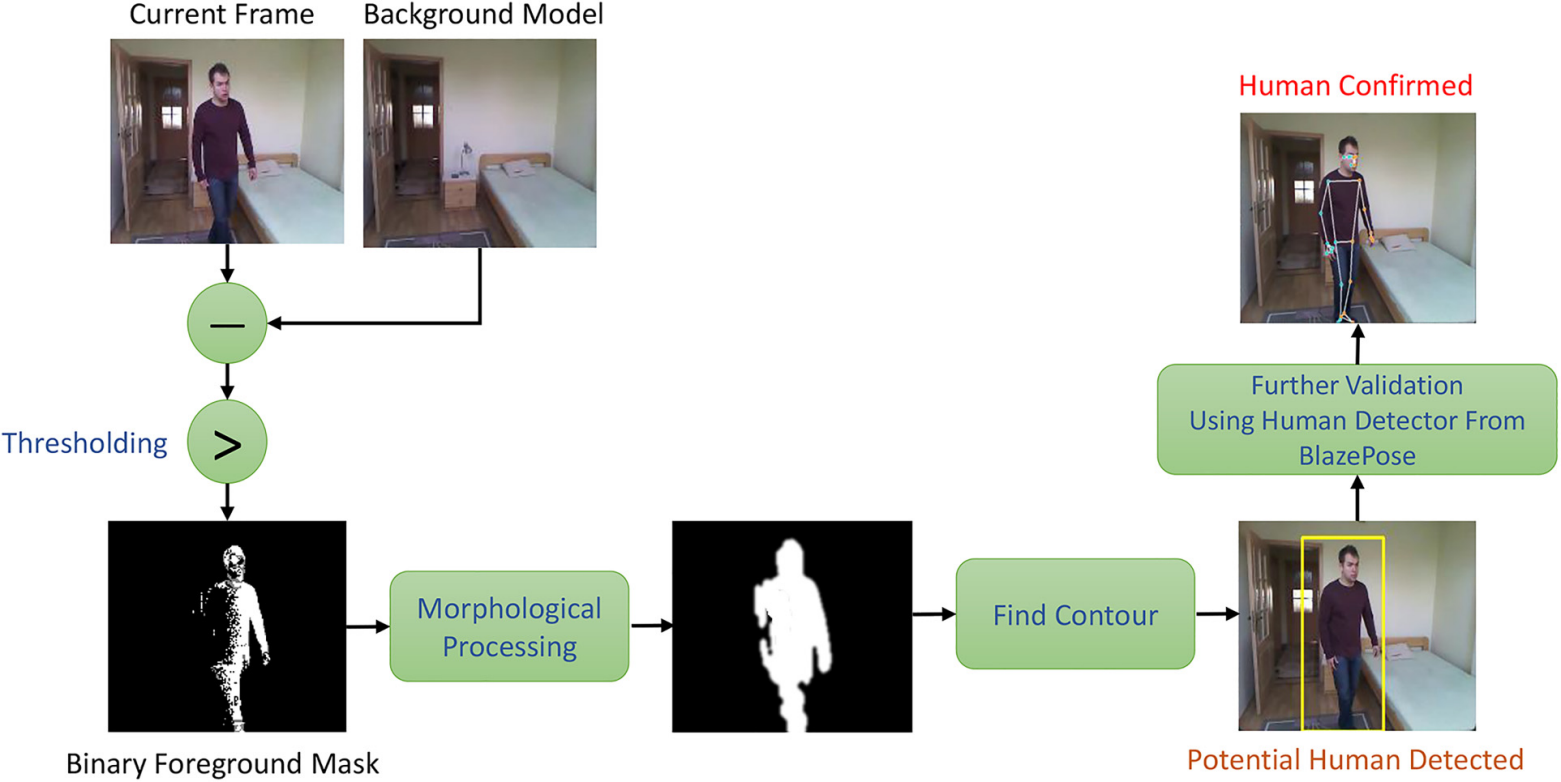
Block diagram of the suggested human detector.

Figure 3 depicts the proposed architecture for human detection. Background subtraction is employed to separate the moving person in the foreground mask from the background model. Following a series of image processing pipelines, a clear view of the body silhouette can be acquired. The designed pipeline includes background subtraction, Otsu's thresholding, morphological processing, Gaussian filtering and contour acquisition. In the end, if the moving object is presumed to be a human, the human detector from the pose estimation neural network BlazePose will further verify the person's presence.

Background subtraction is a technique to isolate moving objects, by determining if a pixel belongs to the background or foreground.^14^ We use kNN-based background subtraction proposed by Zivkovic and van der Heijden^14^ to separate the moving person from the background. This non-parametric algorithm leads to reduced processing time and enhanced segmentation, which aligns perfectly with our objectives.^14^

A greyscale image with shadows is obtained after the background subtraction, so it is necessary to convert the greyscale result into the binary foreground mask for further morphological operations. As a non-parametric method, Otsu's thresholding is highly compatible with the specification of the automated system.^15^ This method searches for the optimised threshold value that minimises the intra-class variance without manual intervention.^15^ The resulting image is transformed into a binary format after thresholding, which serves as the required input for the following morphological operations.

Figure 3 also shows that a rough and incomplete body silhouette has been acquired in the foreground mask after thresholding, which might be caused by light variation and movement irregularity. Dilation and erosion from morphological processing can further enhance the overall representation of the body silhouette.^16^ Intuitively, erosion is used to shrink the thickness of the foreground objects, effectively removing redundant details, while dilation is useful to bridge gaps within the body outline.^16^

After these manipulations above, the contour of the moving object can be simply obtained, and there remains uncertainty regarding whether this object is human. In our proposed method, the contour area determines the initial judgment: if the contour area of the moving object exceeds the threshold value, the detector will indicate that the moving object is potentially a person. Conversely, if the moving object fails to qualify as a human-like object, the system will restart and process a new frame.

In addition, the initial component of BlazePose is a human detection neural network that focuses on precise face detection and predicts body posture.^17^ As the last step of our suggested hybrid human detector, BlazePose's human detector would significantly enhance the confidence of human detection, and it would not excessively compromise the benefit of computational speed from the simplicity of background subtraction.

**Figure 4.**
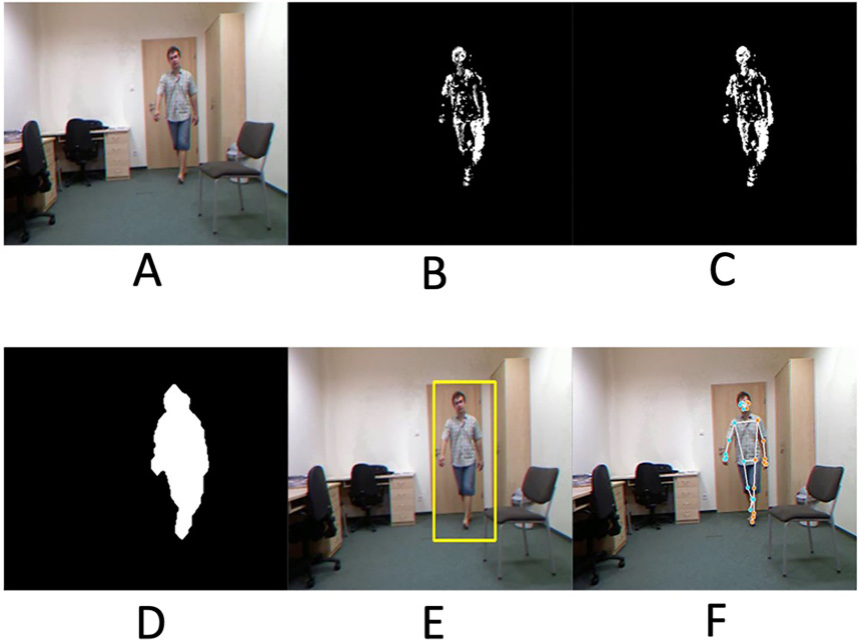
Demonstration of the entire steps in the human detection system: (A) original input frame, (B) background subtraction, (C) Otsu’s thresholding, (D) morphological processing, (E) contouring, and (F) BlazePose's human detection.

Figure 4 goes through all the working procedures of the human detection system, which successfully displays an identifiable body silhouette and detects the presence of a real person.

### Skeleton key point tracker

The first phase of the BlazePose pipeline has been fully utilised to examine the person's presence. In the completed inference pipeline, the following pose tracker is not activated until the human detector returns positive outcomes. Google Research^17^ designed an encoder-decoder architecture to generate a heatmap of the target key points of the body, which is then regressed on the corresponding coordinates by a decoder network. Specifically, the heatmap component is only used in the training phase and can be removed during the coordinate inference, which is the key contribution to significantly decreasing the computational cost. Moreover, BlazePose has sufficiently outstanding performance even if some key points are non-visible or occluded, which increases the confidence of human tracking.

**Figure 5.**
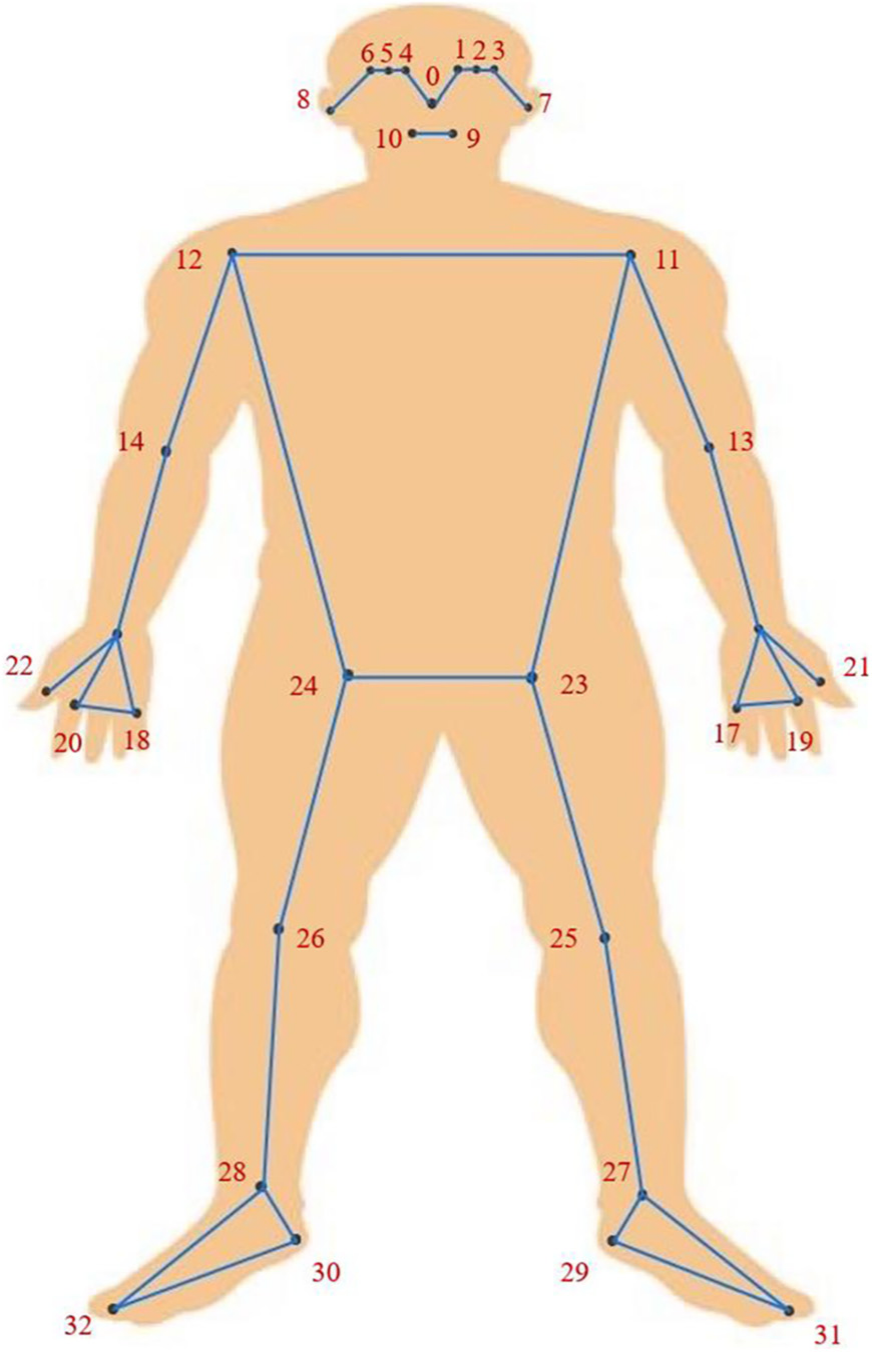
BlazePose's proposed body key point structure.

In contrast to OpenPose, a well-accepted algorithm capable of detecting up to 25 body key points, BlazePose offers a more comprehensive topology of 33 key points, as shown in Figure 5. BlazePose achieves a percentage of correct key points (PCK) with 20% tolerance at 84%, whereas OpenPose shows a slight improvement, increasing the PCK by 1%.^17^ In the videos analysed for this study, BlazePose's operating speed on a laptop CPU is up to 300 times greater than OpenPose's outcome. Besides, a popular pose estimation model MoveNet detects 17 body key points with low latency, but it fails to obtain outstanding results in Alam et al.'s work.^11^ As a result, the pose estimation model BlazePose is more robust and feasible for building up a simple and real-time fall detection system with outstanding reliability.

### Skeleton-based feature engineering

Following the acquisition of accurate human key points, the proposed system algorithm requires the extraction of more specific characteristics as a series of inputs for the final fall recognition algorithm. High-quality features are essential to build a reliable and interpretable fall detection system. Five main features are selected as criteria for fall analysis.

#### Rectangular contour height, width and aspect ratio

Some abnormal activities, such as falls, tend to be accompanied by a substantial change in the human's aspect ratio.^18^ With the help of BlazePose, the estimated width-to-height ratio is able to be expressed as:

(1) $$\begin{array}{rl} w_{\mathrm{contour}}= & \;(\max(\left\{x_{0},x_{1},\ldots ,x_{32}\right\})\\ & -\min(\left\{x_{0},x_{1},\ldots ,x_{32}\right\}))\times W_{\mathrm{frame}} \end{array}$$

(2) $$\begin{array}{rl} h_{\mathrm{contour}}= & \;(\max(\left\{y_{0},y_{1},\ldots ,y_{32}\right\})\\ & -\min(\left\{y_{0},y_{1},\ldots ,y_{32}\right\}))\times H_{\mathrm{frame}} \end{array}$$

(3) $$r=\frac{w_{\mathrm{contour}}}{h_{\mathrm{contour}}}.$$

where *x* and *y* are coordinates normalised by the frame width $W_{\mathrm{frame}}$ and height $H_{\mathrm{frame}}$ , their subscripts symbolise the key point number from Figure 5. All the values experience the de-normalisation to represent the actual lengths, making the algorithm suitable for different scaling of video sequences.

#### Centroid height and vertical velocity

The vertical velocity of the body trunk can be one of the most valuable factors in distinguishing between a fall event and daily living activities.^19^ During a fall event, the effect of gravity leads to the rise of the human's vertical velocity towards the ground.^19^

Here, the centroid height can be simplified to the perpendicular distance between the hip midpoint and the foot midpoint. The vertical velocity can be approximately derived from the change rate of the body's centroid height over a period of time, which can be written as the following:

(4) $$h_{\mathrm{centroid}}=\left(\frac{y_{31}+y_{32}}{2}-\frac{y_{23}+y_{24}}{2}\right)*H_{\mathrm{frame}}$$

(5) $$v_{y}=\frac{h_{\mathrm{centroid}}(f)-h_{\mathrm{centroid}}(f-\Delta n)}{\Delta n}\times N_{\mathrm{video}}$$

where $h_{\mathrm{centroid}}(f)$ denotes the height of the human's centroid at the current frame *f*, Δn represents the frame step for the velocity's measurement, $H_{\mathrm{frame}}$ and $N_{\mathrm{video}}$ refer to the frame height and the video frame rate in frames per second (FPS).

### Cascaded random forests for fall recognition

Random forest is a powerful and effective machine-learning algorithm for regression and classification. It builds up multiple decision trees based on various feature combinations and derives the final prediction based on the results of individual trees.^20^

The algorithm's prediction, when applied to the classification, can be expressed as follows:

(6) $${\hat{C}}_{\mathrm{rf}}^{B}(x)=\mathrm{getMostFrequentElement}\{{\hat{C}}_{b}(x){\}}_{1}^{B}\;$$

where $\hat{C_{b}}(x)$ indicates the prediction of the *b*-th decision tree in the random forest, and we assume there are *B* decision trees in total.

**Figure 6.**
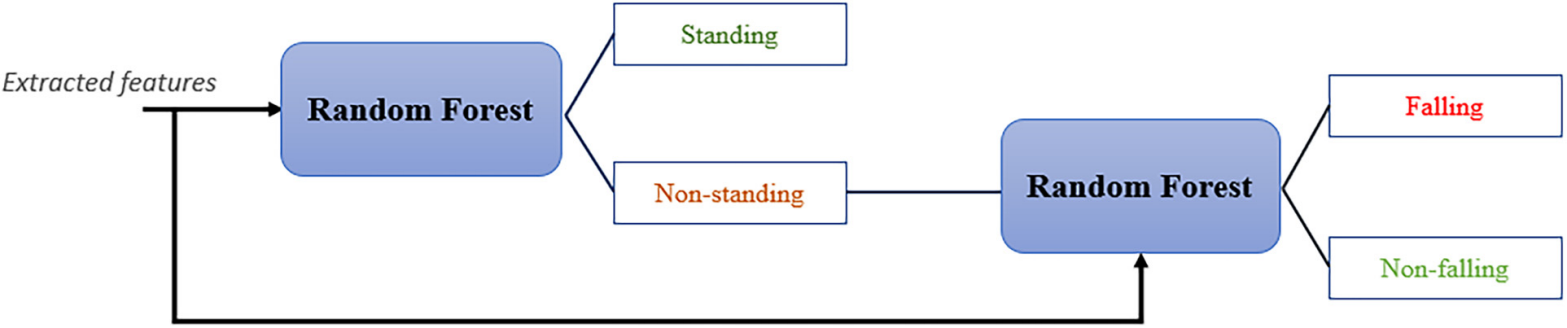
Decision-making flow of the proposed dual-stage cascading random forest classifier.

In our design, the backbone of the designed fall recognition algorithm is a dual-stage cascading random forest structure, depicted in Figure 6. Specifically, both random forests consider all five key features, which include rectangular contour height, rectangular contour width, rectangular contour aspect ratio, centroid height and vertical velocity. These features are selected for their strong correlation with fall-related events. The first stage aims to identify lying, non-lying or temporary poses, and the following random forest determines whether the temporary action should be an actual fall. This multi-layer classifier follows the logic of motion classification and optimises the accuracy of fall judgments.

The proposed architecture for classification is relatively robust to noise because the training phase of the second classifier avoids the interference of data from lying and non-lying events. Also, the random forests are entirely based on the biomechanical characteristics, so great interpretability is one of the design's advantages.

## Results and evaluation

### Experimental setup

**Figure 7.**
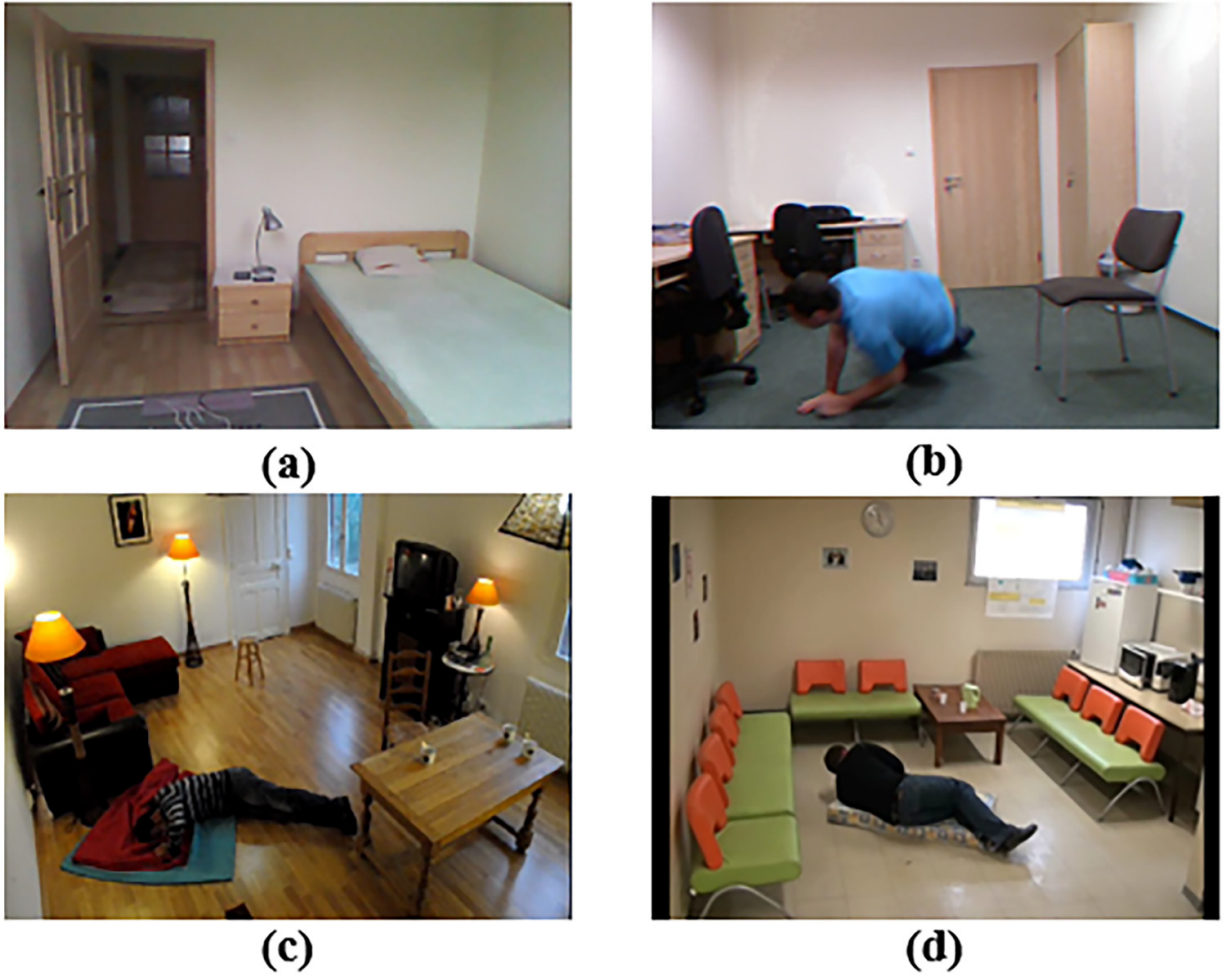
Main scenarios in the dataset: (a) bedroom, (b) office room, (c) living room and (d) coffee room.

We selected the completed UR Fall Detection dataset^5^ and a portion of the Le2i Fall Detection dataset^6^ in our experiments, accompanied by officially annotated labels. By applying our designed scheme, selected features of every RGB frame with a resolution of 640 × 480 are collected to rebuild a new dataset with only numerical data. Then, the size of non-fall samples is reduced to resolve the dataset imbalance and construct a low-bias dataset. After data balancing, there are 10,230 individual frame profiles from the selected dataset: 7163 for training and 3067 for testing and evaluation. The testing set contains a variety of scenes, and the main scenes among them include office room, bedroom, living room, and coffee room, as shown in Figure 7. These scenes exhibit differences in many aspects, including room layout, camera position and lighting conditions.

The proposed algorithm is implemented using Python 3.10, OpenCV 4.7.0 and MediaPipe 0.8.11 on an ordinary laptop with an AMD Ryzen Mobile Processor (Ryzen 7 5800H 3.20 GHz), integrated graphics, and 16 GB RAM.

### Evaluation of classifiers

The designed experiment about the classifier concentrates on the performance for a single frame without consideration of the video context.

In our analysis, we used true positives (TP), true negatives (TN), false positives (FP), and false negatives (FN) to evaluate the model. TP and TN denote the correct prediction of falling and non-falling cases, respectively. FP denotes incorrectly predicted falling, while FN refers to incorrectly predicted non-falling cases. To assess classification performance quantitatively, the accuracy, sensitivity, precision, specificity, and F1 score are calculated as follows:

(7) $$\mathrm{Accuracy}=\frac{\mathrm{TP}+\mathrm{TN}}{\mathrm{TP}+\mathrm{TN}+\mathrm{FP}+\mathrm{FN}}$$

(8) $$\mathrm{Sensitivity}=\frac{\mathrm{TP}}{\mathrm{TP}+\mathrm{FN}}$$

(9) $$\mathrm{Precision}=\frac{\mathrm{TP}}{\mathrm{TP}+\mathrm{FP}}$$

(10) $$\mathrm{Specificity}=\frac{\mathrm{TN}}{\mathrm{TN}+\mathrm{FP}}$$

(11) $$F_{1}=2\times \frac{\mathrm{Precision}\times \mathrm{Sensitivity}}{\mathrm{Precision}+\mathrm{Sensitivity}}.$$

As listed in Table 1, the classifiers achieve an outstanding accuracy of 89.99% on the UR Fall Detection dataset and the Le2i fall detection dataset. And we obtained a sensitivity score of 90.33%. Sensitivity is an essential metric in healthcare applications, which indicates the probability that an actual fall is detected correctly. Low sensitivity will be significantly harmful to the detection outcome of the system.

**Table 1.** Overall performance of the proposed algorithm.

Accuracy	Sensitivity	Specificity	Precision	F1 Score
89.99%	90.33%	89.66%	89.73%	90.02%

We provide further details regarding the system performance in different scenes in Table 2. The accuracy and sensitivity in the four main scenes are consistently around 90%, which proves the high-level generalisation and robustness. Furthermore, the potential versatility improves the practical value of the system.

**Table 2.** System performance in four different scenes.

Scene	Office room	Bedroom	Living room	Coffee room
Accuracy	91.34%	89.19%	90.05%	90.20%
Sensitivity	92.94%	89.95%	88.47%	91.97%

### Algorithm latency analysis

The algorithm latency is of great importance in examining the on-device performance of fall detection. In this section, we will divide the completed algorithm into two components for a comprehensive analysis of its latency: the neural network component for pose estimation and the non-neural network component for human detection and action recognition. We will evaluate the time complexity of the non-neural network part, which assesses how the algorithm's running time grows with input size. Furthermore, we are going to look at the computational cost for the neural network part, usually measured in FLOPs (floating point operations).

In our proposed human detector, we mainly apply a series of digital image processing techniques. Morphological processing and Gaussian filtering generally require relatively higher complexity due to the convolution included. Since the 2D convolution operation can be decomposed into two separated 1D convolutions, the overall time complexity of the digital image processing part is reduced from $O(nk^{2})$ to O(nk) , where *n* is the total number of pixels and *k* is the length of the structural elements or filter kernels. And the computational speed can be further optimised with the support of OpenCV.

Bazarevsky et al.^17^ employs the inference pipeline and compact neural network architecture with only 2.7 million FLOPs in BlazePose. Therefore, we are supposed to expect a substantial decrease in algorithm complexity and computational power requirement.

In our cascaded random forest for action recognition, the time complexity during the prediction can be approximated as O(md) , where *m* and *d* are the number and maximum depth of trees, respectively. Additionally, parallelisation helps the actual implementation of random forest more efficient.

Notably, the proposed algorithm might bypass the BlazePose processing and action classification stages, when no moving person is detected in the scene. This optimisation significantly enhances the overall efficiency of the system as well.

Following the theoretical estimation, we will measure the actual processing time by the human detector and the pose tracker for feature extraction and the actual inference time by the classifier. The processing time specifies the interval between the input of a new frame and the output of the prediction, while the inference time focuses on the time taken for the classifier to acquire the prediction.

Table 3 lists the algorithm latency of our suggested algorithm. Our proposed algorithm can achieve low processing times on the device with limited computational power when the accuracy outcome is maintained at a high level. This outcome verifies our theoretical analysis and shows the powerful real-time performance of our suggested algorithm.

**Table 3.** Analysis of algorithm latency for the proposed system.

Resolution	Computing platform	Inference time per frame	Total processing time per frame	Accuracy	Sensitivity
640 × 480	Ryzen 7 5800H CPU	3.07 ms	26.84 ms (with person)	89.99%	90.33%
			13.00 ms (no person)		

Moreover, we also validate the significance of the hybrid human detector by assessing the improvement in the algorithm's operating speed. We chose all the video sequences that have greater than 2.5 seconds without any person in the scene because these video sequences are more likely to be realistic scenarios and emphasise the value of the hybrid human detector. In this part of the experiment, we first developed a simple algorithm that accomplishes both human detection and pose estimation on the basis of BlazePose. Then we implemented our suggested algorithm with the hybrid human detector. The results related to the operating speed are shown in Table 4. We obtain a real-time on-device operating speed of 29.7 FPS with the hybrid human detector, which results in an improvement of 37.50%.

**Table 4.** Evaluation of real-time performance for the proposed algorithm.

Average time without person per sequence	3.8 s
Total average time per sequence	25.6 s
Operating speed	21.6 FPS (BlazePose only)
	29.7 FPS (Hybrid Detector)
Improvement percentage	37.50%

## Discussion

Four recent works^11–13,21^ are selected for comparative analysis. Khalili et al.'s work^21^ provides detailed interference time of their designed models for a valuable comparison with our findings. We also compare the works related to the real-time fall detection system,^11–13^ which has been reviewed in ‘Related Work’ section. The comprehensive comparison results are summarised in Table 5.

**Table 5.** Comparison of the system performance with recent works.

Evaluated design	Our proposed work	Khalili et al.^21^ (2022)	Alam et al.^11^ (2023)	Lin et al.^13^ (2022)	Qian et al.^12^ (2022)
Sensor	Camera	Camera	Camera	Camera	Gyroscope and Accelerometer
Resolution	640 × 480	320 × 240	256 × 256	–	–
Computing platform	Ryzen 7 5800H CPU	Tesla K80 GPU (12GB)	Ryzen 7 5800H CPU	Two RISC-V CPUs and one KPU	IoT cloud server
Optimisation for the scene without person	Conditional bypass the pose estimation and action classification stages	No	No	No	–
Multiple individuals’ detection	No	No	No	No	–
Inference time Per frame	3.07 ms	–	0.16 ms	–	–
Total processing time per frame	26.84 ms (with person), 13.00 ms (no person)	33 ms (Haar features), 1222 ms (C3D network)	30.52 ms	87 ms	Real-time (via narrowband IoT)
Accuracy	89.99%	95% (Haar features), 99% (C3D network)	84.38%	91.1%	94.88%
Sensitivity	90.33%	89% (Haar features), 96% (C3D network)	92.36%	88.5%	95.25%

Khalili et al.^21^ proposed the weighted training strategy to optimise the accuracy of the fall detection algorithm. They designed Haar feature-based and deep learning algorithms as evaluation. Firstly, features are selected by the AdaBoost algorithm from a large set of Haar features, and then a weighted SVM classifier is then employed for fall recognition. Haar feature-based approach achieved an accuracy of 95%, but the inference time is up to 33 ms on the workstation GPU. Furthermore, they used the 3D neural network (C3D)^22^ to extract the temporal and spatial features and SVM as the final classifier. Although the accuracy rate of 99% was reported, the operating speed exceeded one second for a single input on the workstation GPU, which is unacceptable for a latency-critical application. In addition, the evaluation was restricted to frames with occlusions included, so the overall performance in various scenes was unknown.

Alam et al.'s work^11^ obtained a decent accuracy of 84.38% and real-time operating speed using MoveNet and a threshold-based classifier. Their work measured the accuracy per video sequence, and the use of only 96 video samples was not ideal to derive a robust outcome. Lin et al.^13^ designed a real-time fall detection system based on the AI chip, which can obtain the accuracy of 91.1%. However, the AI chip brings a steep learning curve for users due to its complexity, and the high cost of the AI chip might make this system difficult to be a practical healthcare component. Qian et al.^12^ developed a real-time fall detection framework based on wearable sensor using IoT. The accuracy of 94.88% proves the precision of extracted characteristics, but the latency of IoT and the safety of the sensor were not assessed.

Overall, the proposed system provides an ultra-fast and reliable solution for fall detection. In terms of the operating speed, our algorithm still maintains a real-time performance of around 30 FPS, even based on an affordable AMD laptop processor. In terms of practical value, our system is only dependent on a basic webcam and a budget laptop to achieve non-invasive and contactless detection, which shows more convenience compared to wearable sensor-based methods. In terms of robustness, our algorithm is validated in the different scenes from a large dataset, and we achieve all the accuracies around 90%.

**Figure 8.**
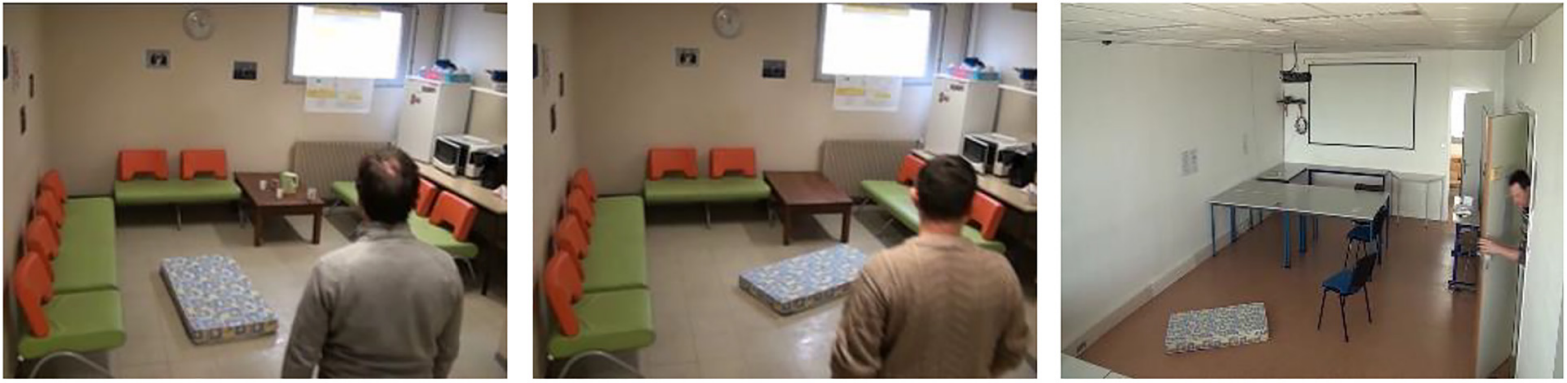
Examples of errors caused by the partial bodies in the frame.

However, there are two limitations to our designed system. The error tends to concentrate on the scene with the partial visibility of the person. For example, only the upper half of a person's body appears in the camera range, as illustrated in Figure 8. This situation surpasses the original capabilities of BlazePose and leads to the extracted information turning into noise in the dataset. In future work, we plan to design an approach to avoid the interference of this complicated situation. Furthermore, the proposed system is unable to achieve multi-person fall detection, as our method is currently optimised for single-person scenarios. In the future, we will explore the possibility of real-time multi-person fall detection algorithms.

## Conclusions

This article proposes a novel vision-based algorithm for reliable and real-time fall detection. In our suggested system, the background subtraction-based human detector decreases the computational cost, and the lightweight pose estimation model BlazePose refines the tracking confidence and feature quality. The conditional execution design and cascaded classifier architecture avoid unnecessary computations and further improve the algorithm's efficiency. We achieved an accuracy of 89.99% and an operating speed of 29.7 FPS on a laptop CPU, similar to the outcome on a workstation GPU of the commonly used fall detection scheme. Our experimental results demonstrate the algorithm's outstanding performance in different scenarios and optimised operating speed in the scene with or without a person. This algorithm shows promise for implementation in portable medical monitoring devices, holding significant implications for elderly care.
